# Linear virtual patients versus student role-plays in teaching patient-centeredness: a mixed-method study

**DOI:** 10.5116/ijme.6a0d.902f

**Published:** 2026-06-18

**Authors:** Michał Pers, Robert Kupis, Andrzej A. Kononowicz, Grzegorz Cebula, Magdalena Szopa

**Affiliations:** 1 Department of Medical Education, Centre for Innovative Medical Education, Jagiellonian University Medical College, Kraków, Poland; 2 Department of Bioinformatics and Telemedicine, Faculty of Medicine, Jagiellonian University Medical College, Kraków, Poland; 3 Department of Metabolic Diseases, Jagiellonian University Medical College, Kraków, Poland

**Keywords:** Patient-centered care, empathy, virtual patients, medical education

## Abstract

**Objectives:**

This study is aimed to assess differences in patient-centered attitudes between students using linear virtual patients focused on the patient’s perspective and those participating in role-play, compare satisfaction with the methods, and explore students’ perceptions.

**Methods:**

A mixed-methods study was conducted among third-year medical students attending a communication course. Students were randomly allocated to either the virtual patient or the role-play group. In total, 122 students participated. Patient-centered attitudes were assessed before and after the intervention using the Patient–Practitioner Orientation Scale. Satisfaction was measured using a 7-point questionnaire. Six students participated in semi-structured interviews. Quantitative data were analyzed using nonparametric tests and equivalence testing, while qualitative were analyzed using thematic analysis.

**Results:**

No differences were observed between the groups in patient-centered attitudes either before or after the intervention. Equivalence testing indicated that outcomes for both teaching methods fell within a predefined equivalence margin of 0.5 points on the Patient–Practitioner Orientation Scale. Students in the role-play group reported higher satisfaction (role-play: median 5.65, IQR = 5.00–6.09; virtual patients: median 5.06, IQR = 4.24–5.62; p = .001). Qualitative interviews revealed that students valued role-play for interpersonal engagement, virtual patients were appreciated for reducing stress and supporting reflective learning.

**Conclusions:**

Methods produced equivalent effects on patient-centered attitudes. Although students expressed a preference for role-play due to its realism, they recognized the reflective and low-stress advantages of virtual patients. Future research should explore hybrid approaches.

## Introduction

Patient-centered care is preferred by patients and, among other factors, improves confidence in physicians, enhances positive lifestyle changes, and reduces symptoms, the need for diagnostic tests, the number of hospitalizations, and treatment costs.^1^^-^^4^ Morgan and Yoder defined the concept by taking into consideration work of several authors as “a holistic (bio-psychosocial-spiritual) approach to delivering care that is respectful and individualized, allowing negotiation of care, and offering choice through a therapeutic relationship where persons are empowered to be involved in health decisions at whatever level is desired by that individual who is receiving the care”.^5^

While there is no doubt about the value of the concept, there are still many challenges in teaching it to medical students. The students complain about modules which are largely focused solely on biomedical knowledge, case studies which are unrealistic and scarcity of positive role models.^6^ Simulated patients are considered the gold standard for teaching communication, but there are limited resources available for using them.^7^^-^^9^ Role-play exercises among students are an older yet cost-effective method that could be a good way to teach communication skills and are noted in literature as an effective tool for improving patient-centeredness among them.^10^^,^^11^

In recent years, virtual patients have emerged as an alternative or supplementary tool in medical education, offering flexibility, reproducibility, and scalability.^12^ Moreover, the increasing diversity of their designs — including linear, branched, and adaptive formats —requires greater clarity in describing which type is studied.^13^ Linear virtual patients, in particular, present clinical scenarios in a fixed, sequential structure, allowing learners to follow a predetermined pathway. They are cost-effective, their cost is constantly falling and they are simple in preparation and the tool is rather easy to use which makes them accessible.^14^ New methods, such as virtual reality, show promise and have received positive feedback, but they are still expensive and require significant resources.^15^

While research on virtual patients is growing, studies focus on their impact on outcomes such as knowledge, skills, satisfaction with only a little insight on their impact on doctor-patient relationship.^14^ Few directly compare the effectiveness of methods in developing patient-centered attitudes, and even fewer specifically target the integration of the patient’s perspective into these modalities, while student attitudes towards virtual patients seem rather positive.^16^^,^^17^ Therefore, their potential for teaching communication skills within patient-centered care has been underexplored.

The aim of this study was to compare the effectiveness and students’ perception of linear virtual patients focused on the patient’s perspective (referred as linear virtual patients during the rest of the study) with student role-play in influencing patient-centered care by changing patient-centered attitudes among undergraduate medical students.

The research objectives included assessing differences in patient-centered attitudes between students using linear virtual patients and role-playing, comparing student satisfaction with the two teaching methods, and exploring students’ perceptions of both methods through qualitative thematic analysis.

## Methods

### Study design

We conducted a mixed-method study combining qualitative and quantitative data to compare linear virtual patients and students role-plays in teaching patient-centeredness. We have decided to use a mixed methods study design to assess differences in patient-centered attitudes as well as students’ experiences between groups using virtual patients and role play in their education.

We enrolled third-year medical students who attended a mandatory, six-meeting communication course. The part of the course are classes focusing on the patient’s perspective. Students are supplied with a short, approximately 15-minute presentation by the teacher, which included definitions of patient-centered care, a concept of patient's perspective as described by Silverman (divided into ideas and beliefs, concerns, expectations and influence on everyday life) which is necessary to deliver patient centered care. In the practical part of this meeting the students are required to role-play three cases involving patients with hypertension, each with the same biomedical background but different perspectives.^18^

### Intervention procedures

We divided student groups into control and experimental groups by a coin toss. The control group was one where the course was organized as planned with no changes, including role-play as a teaching method.

The intervention was to replace the role-plays during the patient’s perspective classes with linear virtual patients created based on the new model.

The model of virtual patients was developed and implemented using the electronic case-based system. Linear virtual patients focused on patient’s perspective were implemented as interactive patient scenarios that presented the patient story following the classical linear HxExIxDxRx structure.^19^ Each of the 3 cases that the students had access to presented the same medical problem of hypertension with similar clinical data related but from three perspectives – of the drug-reluctant patient, of the woman who is hesitating on pregnancy in the future, of the man who is struggling with maintaining a healthy lifestyle. In every case, discovering the patient’s perspective is the key to choosing a treatment the patient will comply to – and non-compliance is a main reason of uncontrolled hypertension.^20^ The virtual cases had the same patient stories as in the role-plays in the control group. They were developed by one of the study authors (MP) and are available as part of the open access clinical reasoning curriculum DID-ACT.^21^

**Figure 1:**
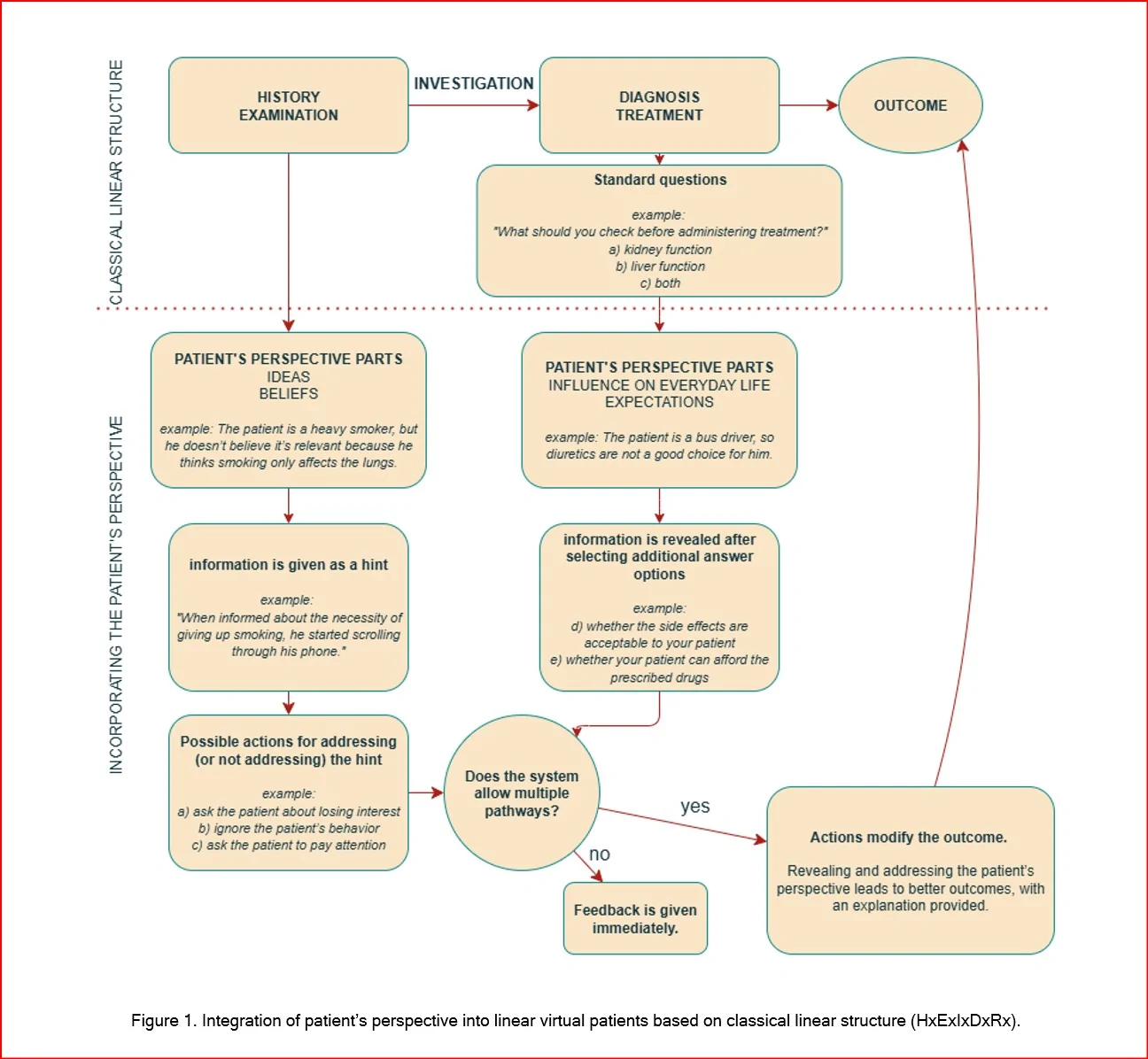
Integration of patient’s perspective into linear virtual patients based on classical linear structure (HxExIxDxRx)

The parts of the patient's perspective were implemented in the virtual patients as described below. Ideas and beliefs, and concerns were integrated into the information-gathering sections, while expectations and impact on life were connected to the parts where the doctor provides information to the patient (see Figure 1).

### Quantitative Measures

According to the ASE (Attitude, Social influence, self-Efficacy) model, human behaviors are the effects of several factors, one of which is attitude. To measure patient-centeredness, defined as patient-centered attitudes, we used the Patient-Practitioner Orientation Scale (PPOS).^22^^,^^23^ The scale is built up of two domains: sharing and caring. It consists of 18 questions. The respondents are asked to answer them using 6-point Likert-scale questions. The final score is calculated as the average from all answers and the higher (the) score is, the more patient-centered the respondent is. We obtained permission from the author of the scale beforehand. The students completed the PPOS before the intervention (at the beginning of the course) and after it (during the last meeting).

To determine students’ satisfaction of the examined method, we used an adapted questionnaire by Cook and colleagues.^24^ The higher the score, the higher the satisfaction, ranging from 1 (definitely disagree) to 7 (definitely agree).

Questionnaire data were collected using LimeSurvey software (LimeSurvey GmbH).

### Qualitative Measures

All students who agreed to participate in the study were invited to join the second part of the study – individual semi-structured interviews. Participants were invited to take part in the interviews three times using the contact information they provided at the beginning of the study. The purpose of this qualitative part was to deepen the understanding of the student perspective to complement the quantitative results from the experimental part of the study. During the interviews, students were asked questions to assess whether they could define patient-oriented care in their own words, what their feelings were about the concept, and whether they saw this concept as important or not. Students were also asked about their feelings and satisfaction with both methods (the interview guide can be found in Appendix). We used the Consolidated criteria for reporting qualitative research (COREQ) checklist to ensure adequate reporting of the qualitative part of the study.^25^

The interviews were recorded, transcribed verbatim and then translated by a private company that anonymized the data. Later, the data were analyzed using six phases of thematic analysis.^26^ The transcripts were analyzed independently by two researchers (MP and RK) and the initial codes were established. Their codes were then discussed and reviewed among the entire research team. The inductive thematic analysis was performed, identifying the main themes and subthemes. All authors accepted the identified themes.

### Participants

The third-year medical students were selected as potential participants due to attending the mandatory course. The students were divided into groups (up to 16 people) by the dean’s office and each group was assigned with the teacher for the course. The teachers were equipped by the authors with all necessary information regarding the study, its aims and the study protocol. During the first out of six meetings students were informed of the study and invited to participate.

The Jagiellonian University Bioethics Committee’s approval was obtained before starting the study (no: 1072.6120.214.2021 granted on 29^th^ of September 2021). All students were reassured that their participation was fully voluntary and that declining it or resigning at any point would have no consequences, including obtaining or not credits for the course. The only exclusion criterium was not obtaining the consent to participate. All data obtained during the study, including transcripts of recordings of the interviews, were anonymized. Due to local bioethical committee restrictions there were no financial incentives for the students for participation in the interviews.

**Figure 2:**
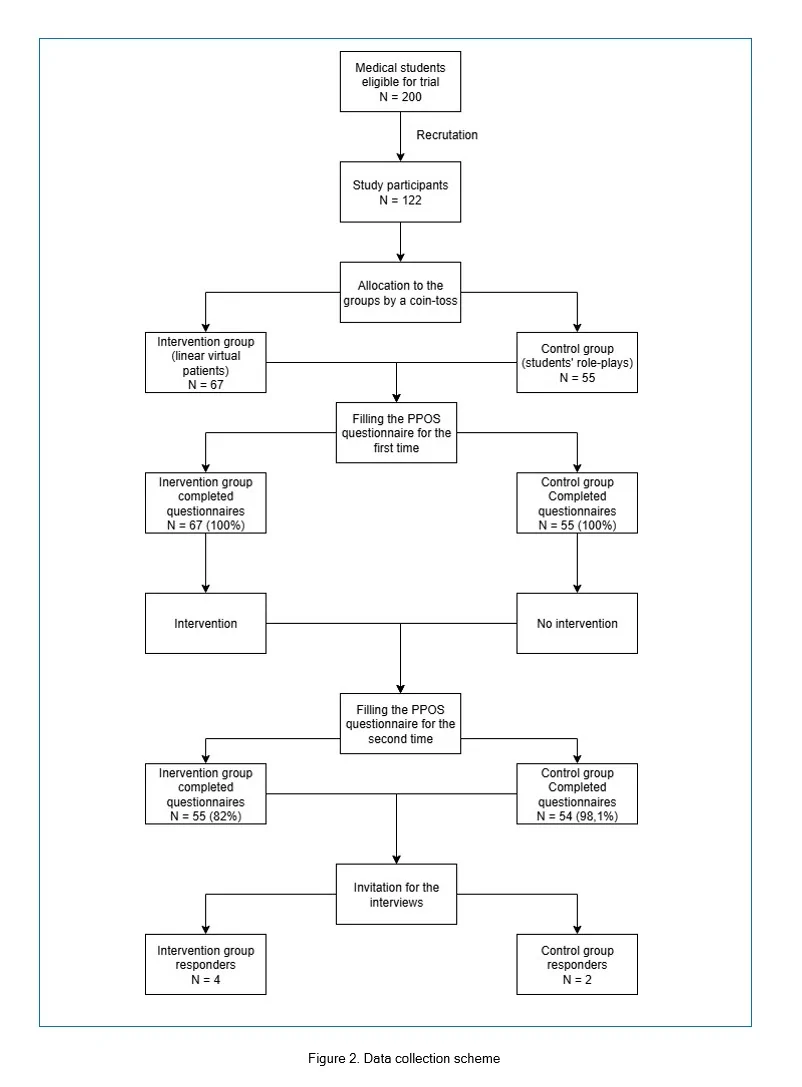
Data collection scheme

The study was conducted between October 2022 and January 2023. We invited students to participate in the interviews after the course, repeating the invitation three times. Six people agreed to take part in the interviews. The data collection scheme is shown in Figure 2.

### Data analysis

Data were analyzed using R statistical software (version 4.3.1; R Foundation for Statistical Computing, Vienna, Austria) and Statistica (version 13.3; TIBCO Software, Palo Alto, CA, USA).

For categorical variables, differences between the experimental and control groups were examined using the chi-square test, with Yates’ continuity correction applied for 2 × 2 contingency tables. Fisher’s exact test was used when expected cell frequencies were low.

For continuous variables, the normality of distributions was assessed using the Shapiro–Wilk test, and homogeneity of variances was evaluated with Levene’s test. As several variables violated the assumption of normality, between-group comparisons were conducted using the Mann–Whitney *U* test.

Associations between quantitative variables were examined using Spearman’s rank-order correlation coefficient. Effect sizes were estimated using Glass’s *Δ*, calculated with the standard deviation of the control group as the denominator. Effect sizes were interpreted as small (< 0.30), moderate (0.30–0.50), or large (> 0.50).

Because a lack of statistically significant differences does not necessarily indicate equivalence, an additional equivalence analysis was performed. The Schuirmann two one-sided tests (TOST) procedure was applied using an equivalence margin of *Δ* = 0.5 points on the PPOS scale. Ninety-percent confidence intervals (*CI*s) for mean differences were calculated, and equivalence was concluded when both bounds of the interval fell within the range from −0.5 to +0.5.

All statistical tests were two-tailed. Statistical significance was evaluated at the .05 level. Descriptive statistics are reported as means (*SD*s), medians (interquartile ranges), and ranges, as appropriate.

## Results

Two hundred third-year medical students were invited to participate in the study. A total of 122 students (61% of the eligible students) participated in the study and filled the questionnaires in the first round.

After the intervention we received 109 questionnaires (89.3% of the number of questionnaires collected in the first round). The group characteristics are shown in Table 1.

There were no statistically significant differences between the groups in demographic characteristics.

**Table 1:** Demographic characteristics of participants before and after the intervention

Variable	Experimental group	Controlgroup	*p*
Before the intervention			
Age, years, median (IQR)	21 (21–22)	21 (21–21.5)	.801
Male, n (%)	27 (40.30)	30 (54.55)	.104
Female, n (%)	39 (58.21)	25 (45.45)	
Nonbinary, n (%)	1 (1.49)	0 (0.00)	
After the intervention			
Age, years, median (IQR)	21 (21–22)	21 (21–22)	.065
Male, n (%)	24 (43.64)	27 (50.00)	.632
Female, n (%)	30 (54.55)	27 (50.00)	
Nonbinary, n (%)	1 (1.82)	0 (0.00)	

Note: Values for age are presented as medians and interquartile ranges (IQRs). Between-group comparisons for age were conducted using the Mann–Whitney U test, and comparisons for sex distribution were conducted using the chi-square test.

### Quantitative analyses

#### The first round

No statistically significant differences were observed between the experimental and control groups in the total score of the Patient–Practitioner Orientation Scale (PPOS) or its subscales (see Table 2). Between-group comparisons conducted using the Mann–Whitney U test yielded non-significant results across all analyses. Effect sizes estimated using Glass’s Δ indicated small effects for all comparisons (|Δ| < 0.30).

#### The second round

A total of 109 completed questionnaires were obtained, representing 89.3% of the questionnaires collected in the first round, including 55 from the experimental group and 54 from the control group. No statistically significant differences were identified between the groups in the total PPOS score or its subscales (see Table 2). Statistical analyses yielded non-significant results, and effect sizes were consistently small (|*Δ*| < 0.30).

### Equivalence analysis

To further examine whether the two teaching methods could be considered equivalent with respect to patient-centered attitudes, the Schuirmann two one-sided tests (TOST) procedure was applied using an equivalence margin of *Δ* = 0.5 points on the Patient–Practitioner Orientation Scale (PPOS). For all PPOS dimensions, the 90% confidence intervals (*CI*s) for the mean differences between the experimental and control groups were entirely contained within the predefined equivalence bounds of −0.5 to +0.5. These results indicate statistical equivalence between the two teaching methods both before and after the intervention (Table 3).

The equivalence findings were further supported by effect size estimates. All Glass’s *Δ* values were below the threshold of 0.30, indicating small effects and supporting practical equivalence between the methods. Specifically, *Δ* values ranged from 0.08 to 0.21 before the intervention and from 0.05 to 0.16 after the intervention.

**Table 2:** Comparison of PPOS Total and Subscale Scores Between Experimental and Control Groups Before and After the Intervention

Variable	Experimental group(n = 67)Median (IQR)	Control group(n = 55)Median (IQR)	*p*
First round			
Sharing	4.00 (3.50–4.31)	3.88 (3.50–4.13)	.24
Caring	4.22 (3.89–4.62)	4.22 (4.00–4.56)	.66
Total PPOS	4.12 (3.85–4.50)	4.06 (3.79–4.32)	.47
Second round			
Sharing	4.00 (3.38–4.50)	3.88 (3.62–4.25)	.44
Caring	4.33 (3.89–4.67)	4.33 (4.00–4.56)	.98
Total PPOS	4.24 (3.76–4.53)	4.09 (3.88–4.29)	.33

### Satisfaction and correlation

A total of 106 students completed the satisfaction questionnaire, including 55 participants from the experimental group and 51 from the control group. The control group reported higher satisfaction levels than the experimental group, as indicated by a higher median score (control group:*median* 5.65, *IQR* = 5.00–6.09; experimental group: median 5.06, *IQR* = 4.24–5.62). The between-group difference was supported by the Mann–Whitney *U* test (*U* = 1023.0, *p* = .001), with a moderate effect size (*r* = .35).

Correlation analyses did not reveal statistically significant associations between satisfaction and any of the Patient–Practitioner Orientation Scale (PPOS) scores, including Sharing (*r* = .15, *p* = .12), Caring (*r* = .12, *p* = .20), and Total PPOS (*r* = .14, *p* = .14).

### Qualitative analysis

Six students (five female and one male), all aged 21, agreed to participate in the interviews. The interviews were conducted via MS Teams by two members of the research team (MP, RK) in February 2023, during the mid-year break to best accommodate students’ availability. The interviews lasted between 8 min 42 sec and 24 min 23 sec. Four students represented the experimental group and 2 – the control group. The qualitative findings were grouped into three main themes: (1) definition and concept of patient-centeredness – that described the students’ perception of what they are being taught. (2) students’ attitudes towards patient-centeredness and the patient’s perspective – that described the perceived outcomes of the intervention. (3) students’ preferences for pedagogical methods used during the classes – that characterize perceptions on how VPs can be incorporated into the CST curriculum in the future. Students are referred as A to F.

**Table 3:** Equivalence Analysis of PPOS total and subscale scores between experimental and control groups using the two one-sided tests (TOST) procedure (Δ = 0.5)

Variable	Experimental groupMedian (IQR)	Control groupMedian (IQR)	90% CI formean difference	*p* (equivalence)
First round				
Sharing	4.00 (3.50–4.31)	3.88 (3.50–4.13)	−0.32 to −0.07	< .001
Caring	4.22 (3.89–4.62)	4.22 (4.00–4.56)	−0.21 to 0.09	< .001
Total PPOS	4.12 (3.85–4.50)	4.06 (3.79–4.32)	−0.24 to 0.06	< .001
Second round				
Sharing	4.00 (3.38–4.50)	3.88 (3.62–4.25)	−0.33 to −0.12	< .001
Caring	4.33 (3.89–4.67)	4.33 (4.00–4.56)	−0.13 to 0.20	< .001
Total PPOS	4.24 (3.76–4.53)	4.09 (3.88–4.29)	−0.20 to 0.14	< .001

### Definition and patient-centeredness concept

When asked about definition of patient-centeredness, all six students pointed out that patient-centered care is an approach where the care and treatment are individualized for the patient. Some of them also included patient’s quality of life, which is not directly mentioned in the definition. However, improving it is the point of delivering the patient-centered care:

“I would say that patient-centered care is one that focuses on anindividualapproach to a given patient and takes into account factors such as the patient's personal beliefs or the things he is afraid of.” (Student A, female, aged 21)

“Focusing on the needs of the individual, on his expectations, feelings, and simply his individual situation. This is a form of providing medical services in which, first of all, we try to help the patient to improve his quality of life, and not to implement specific, rigid procedures. Yes, primarily it is aimed at individualized help for the patient.” (Student E, female, aged 21)

Two persons mentioned the therapeutic relationship and letting the patient be involved in therapeutic decisions:

“All this should be implemented as if it were cooperation with the patient, asking about his expectations, what his goals are in connection with the therapy, his individual goals, such that it has improved his quality of life the most (...). It is important that during the entire therapeutic process you focus on achieving goals that are also important for the patient.” (Student E, female, aged 21)

“That is, they pointed out that the patient's perspective should be taken into account and the treatment should be adjusted accordingly.” (Student F, male, aged 21)

None of the students mentioned all of 4 parts of patient’s perspective but everyone mentioned at least one part of it. All of the parts were mentioned.

“I would say that patient-centered care is one that focuses on an individual approach to a given patient and takes into account factors such as the patient's personal beliefs or the things he is afraid of.” (Student A, female, aged 21)

“[longer thought] Well, in my opinion, this is an approach that is broader than just perceiving the patient as a given case, a given disease, but also perceiving his symptoms, his feelings, and the impact of the disease on his life.” (Student D, female, aged 21)

“First of all, from the beginning, from the beginning of cooperation with a specific patient, both in the interview and during the examination, you should focus on the patient's emotions, on his current knowledge about his health condition and ask about his expectations.” (Student E, female, aged 21)

Regarding the impact of the classes on students' knowledge, there were mixed answers. Three of the students agreed that the course had increased their knowledge on the matter:

“[longer thought] I think so, absolutely. These classes were interesting and I think I learned a lot from them.”(Student D, female, aged 21)

One student admitted that they had been thinking about the concept based on personal experience, which confirmed their own beliefs:

“Well, I guess they didn't change. I mean, I had this approach. I also had experience as a patient, also in hospitals, and I also knew what I expected from doctors, and that's what I had, well, I understood that everyone should be approached individually, so during these classes, it definitely confirmed my belief.”(Student C, female, aged 21)

The rest disagreed:

“I guess I didn't always understand that it would be necessary, needed, that it was important for the patient, right?”(Student B, female, aged 21)

Students’ attitudes towards patient-centeredness and the patient’s perspective

In terms of an attitude towards patient-centered care and including personal perspective into their practice, five students presented positive attitude. They highlighted that patient-centered care may result with increasing patients’ satisfaction with treatment and – secondary – its effectiveness.

“I believe that it is quite important because thanks to this approach we can persuade the patient to undergo treatment, increase his satisfaction with health care and, at the same time, its effectiveness, because a patient who understands the importance of treatment is more likely to comply with it.” (Student A, female, aged 21)

“So it seems to me that there is a great demand to talk about it during studies. And I certainly hope that in my future medical work I will try to implement it in what I do. Well, as I say, I think it is very important.” (Student F, male, aged 21)

One student had a negative attitude towards the concept but admitted that they had started to use obtained skills which had been demonstrated to students during the classes. The student underlined the value of the non-verbal communication.

“I won't say that it made me think any longer, but when it comes to such specifics, I definitely started to learn more, later when collecting interviews and talking to the patient. (...) I definitely started to focus more on asking how much the patient knows at the beginning, to determine the starting point, and also to ask at the beginning about goals, about expectations, to read the patient's facial expression, whether he has any concerns, what his attitude towards a given topic is, and to stop at the right moment and ask at some point what the patient's feelings are up to that point.” (Student E, female, aged 21)

Students were also noticing the practical aspect of using the patient's perspective:

“Perspective can be very helpful, for example, in the sense that you can understand the patient's fears regarding a given procedure, a given treatment, and then simply try to match the arguments and facts to what, for example, the patient is afraid of.” (Student D, female, aged 21)

One student pointed out that using the patient's perspective takes more time during the consultation, which could limit the possibility of using that concept:

“I don't think it's super important to talk about all the patient's emotions and concerns very thoroughly, because it can take up a lot of time that is allotted for a doctor's consultation.”  (Student A, female, aged 21)

Students’ preferences for methods used during the classes

Linear virtual patients group:

One student was strongly against linear virtual patients, which could be connected to technical difficulties.

“Honestly, I didn't like this method (...) there are several reasons. One is that we had technical problems before the class, so it was quite distracting.” (Student A, female, aged 21)

This student also didn’t think that linear virtual patients focused on patients perspective are a good tool for teaching practical skills.

     *(...) I don't think they had a huge impact on our engagement with the patient's perspective.*

This student also believed that the alternative method would be more engaging because of the opportunity to observe the patient’s reaction.

“I think that classes on tablets would be much less effective compared to scenes played by students, because during role-playing we have to be more involved, and besides, I see that observing the other person's reactions, even if they are not so professionally acted, has a much better effect to remember a given experience.” (Student A, female, aged 21)

The other three students were positive towards linear virtual patients, one of them believes that this is a good method of diversification but not a necessity.

[longer thought] “I think it was such an interesting diversification of these classesand I mean, I generally like the interactive additions that we have during classes, so... I think it was useful in this case, but would it be possible without it? wouldn't we make it? I'm sure it would be good too.” [laughter] (Student D, female, aged 21)

But this student also believed that the lack of human interaction makes it not a very good method.

 [longer thought] “Well, in my opinion it's weak, the disadvantage of this method is that there is no interaction with the other person.” (Student D, female, aged 21)

Also, this student believed that role-play is a better method.

 [longer reflection] “I have a little problem with assessing this, but it seems to me that this interaction, this student-student wins here.”(Student D, female, aged 21)

The other student felt more comfortable with the method and thinks that too much stress prevents students from practicing in a thoughtful way.

 “The advantage of this method with tablets over the method with simulated patients is the lower level of student stress. “Personally, I like talking to people, so for me these classes with actors were not very stressful, it was a nice experience. I know that my friends from the group were rather stressed by these types of conversations and then you don't have much time to think about what you're doing, about the purposefulness of certain questions, but you just go to your senses to survive it. However, when there were specific scenarios on these tablets, you could simply notice them calmly, without any stress.” (Student E, female, aged 21)

This student also advised that role-play could be a part of the course but not the only tool.

“Well, I don't think it would replace learning on a live patient, but a system in which such cases on a tablet would certainly be good, such cases would be the first step, and then practicing on live actors.” (Student E, female, aged 21)

The last student believed that role-play gives the opportunity to practice to everyone, not only to the practicing student.

“That the most effective element of such work on a tablet is that we have to fully experience some scenario ourselves and, well, it is quite valuable. So far, it's rare at university that we have to work through a problem ourselves from start to finish, so these classes on tablets allow for so much freedom.” (Student F, male, aged 21)

### Role-playing group:

One student wasn't entirely convinced that role-play is superior to linear virtual patients

“And I don't know. [laughter] I guess it depends on the students, because there are different students and most of all they like it, no, making such scenes in front of other friends, I don't know. It's different, I think so.” (Student B, female, aged 21)

But this student wasn’t entirely convinced that linear virtual patients are superior either.

[longer thought] “I guess we should try this and this and this. [laughter] And then compare. I think why is it worse? No, it won't be worse.” (Student B, female, aged 21)

The second student strongly advocated for role-play.

“I think so, because the person who plays the role of the patient must also focus a little to actually convey any specific concerns to the doctor, or simply, like, well, it seems to me that ... [longer thought] Honestly, it seems to me that it is probably less effective, because it is an interaction with a living person.” (Student C, female, aged 21)

### Integration of Findings

The integration of quantitative and qualitative findings provides a more comprehensive understanding of how linear virtual patient simulations and role-play sessions supported the development of patient-centered attitudes among medical students.

Quantitative analyses revealed no statistically significant differences between groups in patient-centeredness, as measured by the PPOS total score and its subscales, across both rounds of assessment. Equivalence testing confirmed that both instructional methods achieved comparable outcomes within a predefined margin (Δ = 0.5).

Students’ qualitative feedback and satisfaction ratings offered additional context for interpreting these results. Although participants in both groups reported increased awareness of the patient’s perspective and communication processes, those in the role-play group expressed higher satisfaction with the learning experience, emphasizing the realism, emotional engagement, and interpersonal aspects of direct interaction. In contrast, students in the linear virtual patients group valued the structured, low-pressure environment that allowed them to focus on reasoning and reflection.

Together, these findings indicate that both methods foster similar attitudinal outcomes through distinct learning mechanisms: role-plays through emotional and interpersonal engagement, and linear virtual patients through structured cognitive reflection. The complementarity of these approaches suggests that integrating both modalities within the curriculum may enhance the overall development of patient-centered communication skills in medical education.

## Discussion

This study addressed a key gap identified in the existing literature on the use of linear virtual patients for teaching patient-centered care. While previous research has primarily focused on the impact of virtual patients on clinical reasoning, diagnostic accuracy, or procedural knowledge^12^^,^^16^^,^^17^, much less attention has been given to their potential in shaping patient-centered attitudes and communication skills — domains traditionally supported by interactive methods such as role-play or simulated patients.^8^^-^^10^. Moreover, few studies have directly compared linear virtual patients, the most accessible and cost-effective type of virtual patients, with student role-play in this context.

Our mixed-method study was designed to explore whether linear virtual patients can achieve comparable attitudinal outcomes to role-plays while providing insights into students’ learning experiences with both methods. Quantitative analyses using the Patient–Practitioner Orientation Scale (PPOS) showed no significant differences between groups in either round of measurement. Equivalence testing suggested that both methods did not differ in influencing patient-centered attitudes. These results support earlier observations that diverse experiential learning strategies can cultivate empathy and patient-centeredness in medical education.^27^

Patient-centeredness in general was positively received by the students. However, none of them was able to recall all aspects of patient-centered care, although all provided an understandable definition and could explain, in simple terms, the general concept. Some highlighted that patient-centered care may improve patients’ quality of life. Even when students expressed reservations about patient-centered care, they acknowledged its usefulness when conducting medical interviews or breaking bad news to patients. On the other hand, although they had limited clinical experience, many noted that patient-centeredness might be difficult to implement in everyday practice because it is time-consuming. Contrary to the findings of Henschen et al. regarding students’ perception of patient-centered care, participants in our study were able to confront the theoretical background of the paradigm with practice during classes. Nevertheless, further training in clinical settings would be beneficial.^28^

Students often tend to adopt a more doctor-centered approach and demonstrate relatively low attitudes toward patient-centered care.^29^ From the personal experience of some of the study authors as course instructors, many students report that the communication skills and patient-centered approaches they are taught during preclinical classes sometimes do not align with what they observe during clinical rotations. This observation is consistent with the findings of Stratta et al. regarding empathy erosion among medical professionals.^30^ Such polarization creates space for further pedagogical intervention — not only among students but also within the teaching faculty. The authority of experienced care providers does not necessarily make this task easier. Clinical role models encountered by students are often physicians who operate in productivity-centered environments, leaving little room for nurturing patient-centered behaviors learned during studies. Over time, without awareness and institutional support, this dynamic may contribute to physician burnout and reduced quality of patient care.^31^ Fortunately, communication training has been shown to mitigate the risk of empathy erosion.^32^

The qualitative data collected in our study sheds light on the perspectives of the youngest generation of future healthcare providers. Although limited, the emerging themes may provide a more nuanced understanding of students’ attitudes toward the physician–patient relationship and patient-centered care.

Our findings revealed that students were rather critical of virtual patients, pointing out drawbacks of this teaching method for patient-centeredness training. However, they also proposed a solution, suggesting that virtual patients could serve as a supplement to role-playing. This indicates that students value contact with real patients and recognize its unique contribution to learning. Lorenzini et al. suggest that, given the growing role of artificial intelligence (AI) and technology in medicine, the current paradigm of shared decision-making should be redefined to include the doctor, the patient, and AI.^33^ Properly designed AI-assisted decision-making may help to ensure that patients’ preferences, beliefs, and values are respected, thereby preserving their autonomy. Ultimately, AI-supported collaboration could enhance care quality. Nonetheless, introducing AI into clinical settings also carries new risks, including AI-driven paternalism.^34^ At the undergraduate level, however, virtual patients may support clinical and communication training as a relatively inexpensive, accessible, and flexible tool, enabling interactive and self-directed learning for all students.

The integration of quantitative and qualitative findings provides a deeper understanding of how each method supports patient-centered learning. Both groups reported increased awareness of the patient’s perspective and the interpersonal aspects of communication, yet qualitative feedback revealed distinct learning mechanisms. Students participating in role-play emphasized realism, emotional engagement, and direct interpersonal interaction as central to their learning, whereas those using linear virtual patients appreciated the structured, reflective environment that reduced performance anxiety and allowed them to focus on decision-making and perspective-taking. These findings align with previous studies suggesting that active reflection and cognitive engagement can reinforce attitudinal change even in less interactive simulation formats.^18^^,^^26^^,^^27^

Although both groups achieved comparable PPOS scores, students engaged in RP reported significantly higher satisfaction with the learning experience. This contrast underscores the multifaceted nature of student engagement: while both methods may equally support attitudinal outcomes, role-play appears to provide a stronger affective and motivational dimension, which contributes to perceived learning value. Similar results have been reported in previous studies, where students valued the emotional immediacy of interpersonal practice even when objective outcomes were comparable.^6^^,^^28^^,^^32^

Taken together, these results indicate that linear virtual patients and role-plays foster similar levels of patient-centered orientation through distinct but complementary mechanisms — cognitive reflection in linear virtual patients and emotional immersion in role-plays. This complementarity supports the pedagogical rationale for combining structured, technology-enhanced learning tools with interactive interpersonal training. Such an integrative approach could optimize both the reflective and empathic dimensions of communication training in medical education.^35^

Our study is not free from limitations. First, the results come from a single center; therefore, further studies are needed to generalize the findings. Although we did not demonstrate an improvement in patient-centered attitudes, this is understandable since attitudinal change is inherently challenging, and the method we compared (role-plays) is already widely used in communication skills training. Second, the sample size, particularly the number of interviewed students, was relatively small. Due to local bioethical regulations, we could not offer any incentives for participation, and recruitment occurred during a busy academic period. To achieve full data saturation, future research should include a larger and more diverse cohort. Third, the outcomes of the virtual-patient classes might have been influenced by occasional technical issues encountered during sessions. Ensuring smooth technical performance is crucial for maximizing learning outcomes, although minor glitches are often unavoidable. Furthermore, qualitative findings may be biased, as students who volunteered for interviews might have been more articulate or motivated to share their perspectives. Including students at various stages of training could provide a more comprehensive picture. Finally, the PPOS was translated according to WHO guidelines but was not validated prior to use. All these aspects highlight areas for further investigation.

In summary, this study helps to fill a critical gap in the understanding of how virtual patient tools can be used to promote patient-centeredness in medical education. Previous work has predominantly emphasized cognitive learning outcomes, with little empirical evidence regarding attitudinal development. By directly comparing linear virtual patients and role-plays, our findings extend the pedagogical value of virtual patients beyond cognitive domains, highlighting their potential as scalable tools for fostering reflective, patient-centered thinking. Future research should further explore hybrid educational models that combine emotionally engaging simulations with structured virtual scenarios to maximize their impact on patient-centered communication and professional identity formation among medical students.

## Conclusion

Our findings have shown no difference between linear virtual patients focused on patient’s perspective and students’ role-plays in effects on patient cantered attitudes. Students were significantly more satisfied with role-plays than virtual patients.

Qualitative analysis has shown that students understand the concept of patient’s perspective, they feel more positive about role-plays but see the positives of linear virtual patients and even suggesting mixing the methods as most effective way of using them.

Since role-plays are one of the standard methods of teaching clinical communication these findings suggest that the idea that linear virtual patients focused on patient’s perspective could serve as a substitute when holding real-time classes is not possible or when a student is absent and needs a way to make up the classes. It is also possible to mix the methods during the classes to make them more engaging for students.

We cannot recommend linear virtual patients focused on patient’s perspective as the sole alternative method of conducting classes since satisfaction was significantly higher in the role-play group.

### Acknowledgments

We thank the students who participated in this study.

### Conflict of Interest

The authors declare that they have no conflict of interest.

## Supplementary material

Supplementary file 1 Appendix. The interview guide used during collecting data
