# Supplementary material for: Linear virtual patients versus student role-plays in teaching patient-centeredness: a mixed-method study
Source: Int J Med Educ. 2026 Jun 18;17:77–89. doi: 10.5116/ijme.6a0d.902f (PMC13563274; doi:10.5116/ijme.6a0d.902f)
Supplement: Supplementary file 1 [file ijme-17-77-S1.pdf]

Appendix

The interview guide used during collecting data

- How would you define patient-centered care after completing the class?
- Did the class influence your knowledge on this topic?
- What are your thoughts on patient-centered care after the class?
- Do you think the class encouraged you to reflect on patient-centered care?
- What is your attitude towards the patient's perspective – that is, information about their emotions, concerns, and needs? In your opinion, how important is this type of information gathered from the patient?
- Has your approach to the patient's perspective – that is, information about their emotions, concerns, and needs – changed after completing the class? If yes, in what way? If not, why not?
- Do you consider the method used during the class effective in encouraging reflection on patient-centered care and the patient's perspective? Please address both aspects separately.
- In your opinion, what was particularly effective about this method?
- In your opinion, what are the weaknesses of this method?
- Do you think an alternative method (e.g., role-play or virtual cases) would be less or more effective in encouraging reflection on person-centered care and the person's perspective? Please address both aspects separately.
